# Association of low-grade inflammation caused by gut microbiota disturbances with osteoarthritis: A systematic review

**DOI:** 10.3389/fvets.2022.938629

**Published:** 2022-09-12

**Authors:** Wu Xiang, Bingjin Ji, Yiqin Jiang, Han Xiang

**Affiliations:** ^1^Department of Rehabilitation, Beibei Traditional Chinese Medical Hospital, Chongqing, China; ^2^Department of Radiology, Daping Hospital, Army Medical University, Chongqing, China

**Keywords:** low-grade inflammation, gut microbiota, gut microbiota disturbances, osteoarthritis, systematic review

## Abstract

**Background:**

Currently, many studies have been published on the relationship between the gut microbiome and knee osteoarthritis. However, the evidence for the association of gut microbiota with knee osteoarthritis has not been comprehensively evaluated.

**Objective:**

This review aimed to assess existing results and provide scientific evidence for the association of low-grade inflammation caused by gut microbiota disturbances with knee osteoarthritis.

**Methods:**

This study conducted an extensive review of the current literature using four databases, PubMed, EMBASE, Cochrane Library and Web of Science before 31 December 2021. Risk of bias was determined using ROBINS and SYRCLE, and quality of evidence was assessed using GRADE and CAMADARES criteria. Twelve articles were included.

**Results:**

Studies have shown that a high-fat diet leads to a disturbance of the gut microbiota, mainly manifested by an increase in the abundance of Firmicutes and Proteobacteria, a decrease in Bacteroidetes, and an increase in the Firmicutes/ Bacteroidetes ratio. Exercise can reverse the pattern of gain or loss caused by high fat. These changes are associated with elevated levels of serum lipopolysaccharide (LPS) and its binding proteins, as well as various inflammatory factors, leading to osteoarthritis (OA).

**Conclusion:**

This systematic review shows that a correlation between low-grade inflammation caused by gut microbiota disturbances and severity of knee osteoarthritis radiology and dysfunction. However, there was a very small number of studies that could be included in the review. Thus, further studies with large sample sizes are warranted to elucidate the association of low-grade inflammation caused by gut microbiota disturbances with osteoarthritis, and to explore the possible mechanisms for ameliorating osteoarthritis by modulating gut microbiota.

## Introduction

Osteoarthritis (OA) is the most common musculoskeletal disease and one of the leading causes of disability (1). Epidemiological surveys show that more than 320 million people worldwide suffer from OA, and the prevalence is higher in women than men. Traditionally, mechanical and genetic factors have been considered important causes of OA (2, 3). However, emerging evidence suggests that low-grade inflammation plays an important role in the development of OA (4), and this inflammatory state is closely related to the gastrointestinal microbiota (5).

The gastrointestinal microbiota refers to the sum of all genetic material and its metabolites of all microbiota present in the gut (6, 7). The gut microbiota plays an important role in maintaining the body's homeostasis, which underlies human physiology, immune system development, digestion, fat storage, regulation of angiogenesis, behavior, development, and detoxification responses. The human gut microbiota is mainly composed of Firmicutes, Bacteroidetes, Actinobacteria, Proteobacteria and Verrucobacterium. Among them, Bacteroidetes and Firmicutes account for more than 98% of the total number of intestinal symbiotic flora of more than 70 species (8, 9). Studies have shown that a variety of diseases are associated with specific bacterial sequences and alterations and disturbances in the composition of the microbiota (10, 11). At the same time, the gut microbiota plays a key role in the development and function of the immune system, as well as in allergic and inflammatory responses (12–15). Alterations in the microbiome activate the innate immune system, leading to increased pro-inflammatory cytokines, and these local and systemic low-grade inflammations contribute to the development and progression of OA (16, 17).

At present, there are more and more studies on the correlation between low-grade inflammation caused by intestinal flora disturbance and OA. It is difficult to draw conclusions about the consistency of the association due to different study designs and assessment methods, so it is unclear whether low-grade inflammation due to disturbances in the gut microbiota has a different effect on OA. Given the high prevalence of OA and its significant socioeconomic burden, it is important to explore the impact of low-grade inflammation caused by gut microbiota disturbances on OA.

## Methods

### Search strategy

We searched comprehensively for articles published before 31 December 2021 using four electronic medical databases (PubMed, EMBASE, Cochrane Library and Web of Science). Studies were identified using the search terms “('gut microbiota' or 'microbiome' or 'microbiota' or 'gut') and ('Osteoarthritis' or 'arthritis' or 'KOA' or 'OA') and ('Inflammation')”.

### Selection criteria

Inclusion criteria: (1) clinical and basic research with any level of evidence; (2) English-language articles published in peer-reviewed journals; (3) studies on the association of low-grade inflammation caused by gut microbial imbalances with OA, and OA Pathogenesis or related-symptoms. Exclusion criteria: (1) studies with missing data; (2) studies with duplication and poor scientific method; (3) abstracts, case reports, conference reports, reviews, editorials, and expert opinions were excluded.

### Literature screening and data extraction

Two investigators (WX and HX) independently searched, selected relevant articles according to the inclusion and exclusion criteria, read the full text, and extracted data from the final included literature. Any disagreements were resolved by an experienced systematic reviewer (BJJ). Differences in data extraction are resolved by consensus.

**Figure 1:**
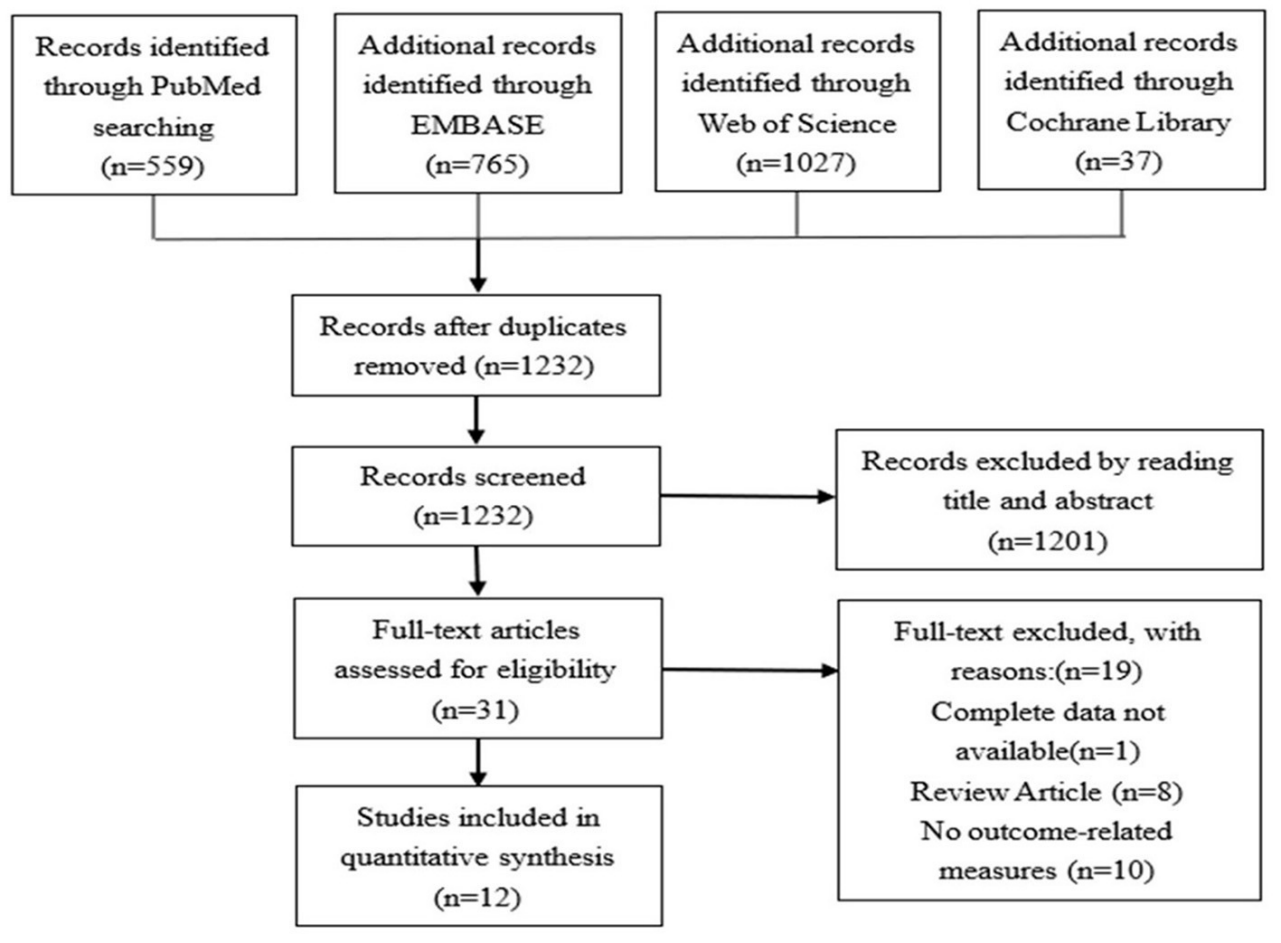
Literature search and screening flowchart.

After extraction, the data was considered of heterogenous nature both by study design, measure, and method of assessment. Therefore, a descriptive analysis approach was preferred to a metanalysis. Figure 1 for details.

### Risk of bias assessment

ROBINS was used to assess the risk of bias in non-randomized clinical studies (18), and RoB 2.0 (19) was used to assess the risk of bias in randomized clinical studies. Risk of bias in preclinical studies was assessed using SYRCLE (20). WX and HX conduct evaluations independently, and any disagreements are resolved by consensus.

### Study quality assessment

The quality of clinical studies (*n* = 6) was assessed using the GRADE method (21) and each study was classified as 'low', 'moderate' or 'high'. All studies were ranked 'moderate' or 'high'. The quality of preclinical studies (*n* = 6) was assessed using the Collaborative Approach to Meta-Analysis and Review of Animal Data from Experimental Studies (CAMADARES) checklist (Supplementary material) (22, 23). Each study was scored on a scale from 0 to 10 points, and the overall quality of included studies was moderate (mean CAMADARES score 4.17, range 4–5). WX and HX conduct evaluations independently, and any disagreements are resolved by consensus.

## Results

### Study characteristics

The final analysis included 12 studies, 6 of which were animal studies (24–29) and 6 were clinical studies (5, 30–34). Regarding clinical trials, 4 were non-randomized observational studies (5, 30, 33, 34) and 2 were randomized clinical trials (31, 32). The main characteristics of the included studies are reported in Table 1.

**Table 1:** The main findings of the included studies.

**Source**	**Total No**.	**Sex**	**Age Mean(SD)**	**Assessment of OA**	**Assessment of Inflammation**	**Main Findings**
Huang et al. (30)	25 humans	18 female	62.4(15.8)	JSN score, NHANES-I and WOMAC score	LPS and LBP	LPS is important in the pathogenesis and severity of KOA.
Lei et al. (31)	461 humans	241 female	Lcs group: 66.5(5.2) Placebo group: 67.2(4.8)	WOMAC score and VAS score	hs-CRP	LcS can improve OA by reducing serum hs-CRP levels.
Huang et al. (32)	431 humans	all female	54.7(5.64)	uCTX-II, JSW and JSN	LBP, sTLR4 and IL-6	Plasma LBP and sTLR4 correlate with KOA progression, suggesting a role for systemic low-grade inflammation in KOA pathogenesis.
Ulici et al. (24)	50 mice	all male	Younger 12-18 weeks, older 37-48 weeks	ACS, osteophyte size and synovial hyperplasia	IL-6, LPS and LBP	Factors related to the gut microbiota promote the development of OA after joint injury.
Boer et al. (5)	1427 humans	821 female	56.9 (5.9)	knee WOMAC pain scores and Kellgren-Lawrence radiographic OA severity scores	the amount of effusion of knee	Abundance of Streptococcus species is associated with increased knee pain and this association is driven by local inflammation in the knee joint. The microbiome is a possible therapeutic target for KOA.
Huang et al. (26)	42 mice	6 male and 6 female	8 weeks	OARSI score, Safranin O score and Synovitis score	G-CSF, IL-1β, IL-6, IL-10, IL-17, IP-10, MCP-1, MIP-1α and LPS	Changes in the gut microbiota can promote the development of OA.
Guan et al. (25)	54 mice	27 male and 27 female	8 weeks	DXA, Micro-CT and OARSI score	MMP-13	Antibiotic-induced gut dysbiosis reduces serum lipopolysaccharide levels and inflammatory response, resulting in decreased MMP-13 expression and improved OA.
Jhun et al. (27)	36 mice	male rats	6 weeks	modified Mankin score and Matrix staining	IL-1β, LPS, MCP-1, CCR-2, PPAR-γ, GABA, MMP3, TIMP1, TIMP3, SOX9, COL2A1 and IL-10	Intestinal damage and inflammation were improved by L. rhamnosus and own the therapeutic potential in OA.
Li KF et al. (28)	54 mice	all male	8weeks	Mankin score and cartilage thickness	LPS, TLR-4 and MMP-13	Exercise can relieve of OA and chronic inflammation, which is a potential therapeutic way for obesity-related OA.
Dunn et al. (33)	75 humans and 23 mice	48 women and 23 male mice	11 weeks	OARSI score	LPS and LBP	Reveal a microbial DNA signature in human and mouse cartilage and identify strain-specific signatures within mouse cartilage that mirror human patterns.
Won et al. (29)	mice	NA	12 weeks	OARSI score, synovitis score and Osteophyte size	TLR-2, TLR-4, LBP and CD14	LBP and CD14 are necessary for the exacerbation of posttraumatic OA cartilage destruction resulting from low-grade inflammation.
Loeser et al. (34)	92 humans	69 female	Cases 73.7(6.9) and controls 70.8(6.4)	WOMAC pain score, AUSCAN hand pain score, ACS score Safranin-O score and osteophytes	LPS and LBP	The increasement of serum LPS levels may contribute to development of OA associated with obesity.

uCTX-II, urinary (u) C-telopeptide of Type II collagen; JSW, radiographic tibiofemoral joint space width; JSN, joint space narrowing; WOMAC, Western Ontario McMaster Universities; VAS, Visual Analog Scale/Score; LPS, lipopolysaccharide; LBP, lipopolysaccharide-binding protein; TLR2, Toll-like receptor 2; sTLR4, soluble Toll-like receptor 4; TLR4, Toll-like receptor 4; IL-1β, interleukin-1β; IL-6, interleukin-6; IL-10, interleukin-10; IL-17, Interleukin 17; MCP-1, Monocyte chemoattractant protein-*1*; CCR2: Recombinant Chemokine C-C-Motif Receptor 2; MMP3, matrix metallopeptidase 3; MMP-13: matrix metalloproteinase-13; GABA, γ-aminobutyric acid; PPAR-γ, peroxisome proliferator-activated receptor γ; TIMP1, tissue inhibitor of metalloproteinases *1*; TIMP3, tissue inhibitor of metalloproteinases *3*; MLI, Meniscal/Ligamentous Injury; METS, Metabolic Syndrome; OA, Osteoarthritis; KOA, knee Osteoarthritis; ACS, scores for articular cartilage structure; Lcs, Lactobacillus casei Shirota; hs-CRP, high-sensitivity C-reactive protein; OARSI, the Osteoarthritis Research Society International; GCSF, Granulocyte-colony stimulating factor; IP-10, Interferon Gamma-Induced Protein 10; MIP-1α, Macrophage Inflammatory Protein 1α; CD14, cluster of differentiation 14; AUSCAN, The AUStralian CANadian Osteoarthritis Hand Index;SOX9, SRY-related high mobility group-box gene9; COL2A1, Type II collagen fiber α1 gene; DXA, Dual Energy X-ray Bone Densitometry.

Most studies used 16S ribosomal RNA (rRNA) gene sequencing to examine gut microbiota and Enzyme-linked immunosorbent assay (ELISA) to measure inflammatory markers. Meanwhile, most studies assessed radiographic or symptom severity of OA using Western Ontario McMaster Universities (WOMAC) score, Visual Analog Scale (VAS) score, scores for articular cartilage structure (ACS) score, the Osteoarthritis Research Society International (OARSI) score, synovitis score and Osteophyte size. Overall, various studies have suggested that there is a certain relationship between inflammation caused by intestinal flora disturbance and OA.

### Effects of diet, exercise or probiotics on gut microbiota

High-fat diet leads to gut microbiota disturbances and is a common model of low-grade inflammation (35). Firmicutes, Bacteroidetes and Proteobacteria are the three major phyla of the gut microbiota (28). High-fat diet cause disturbance of the gut microbiota, increase endotoxin-producing bacteria, and decrease bacteria protecting the intestinal barrier, thereby enhancing bone destruction on OA in mice. It is mainly manifested by an increase in the abundance of Firmicutes and Proteobacteria, but a decrease in Bacteroidetes, and an increase in the Firmicutes/Bacteroidetes ratio (28).

Exercise reverses high fat diet-induced gut microbiota disturbances, manifested by decreased abundances of Firmicutes and Proteobacteria, increased abundance of Bacteroidetes, and decreased Firmicutes/Bacteroidetes ratios. At the family level, exercise reversed the unclassified Bacteroidetes, Lachnospira, Desulfovibrio, Ruminococci, Lactobacillus, Prevotaceae, Peptostreptococcus, Bifidobacterium, and Staphylococcus (28).

Two studies suggest that probiotic supplementation reduces intestinal damage and inflammation, and has great potential in the treatment of osteoarthritis (27, 31).

### The influence of intestinal flora disturbance on OA

Intestinal microbial disturbances increase intestinal permeability and cause low-grade inflammation throughout the body, thereby aggravating OA. By transplanting human microorganisms into mice, it was found that the abundance of Fusobacterium and Enterococcus faecalis in the transplanted mice increased, but the abundance of Ruminococcus decreased, the average systemic concentration of inflammatory markers increased, and the intestinal increased permeability is associated with more severe OA (26). At the same time, the serum estrogen level in OA rats was significantly decreased, which was correlated with the significant increase in LPS. In Lactobacillus rhamnosus-treated OA rats, the expression levels of Monocyte chemoattractant protein-*1 (*MCP-1*)* and its receptors Recombinant Chemokine C-C-Motif Receptor 2 (CCR2), interleukin-1β (IL-1β), matrix metallopeptidase 3 (MMP3) were decreased, while γ-aminobutyric acid (GABA) and peroxisome proliferator-activated receptor γ (PPAR-γ), tissue inhibitor of metalloproteinases 1 (TIMP1), tissue inhibitor of metalloproteinases 3 (TIMP3), SRY-related high mobility group-box gene9 (SOX9) and Type II collagen fiber α1 gene (COL2A1) and interleukin-10 (IL-10) increased expression levels (27).

### The effect of inflammation on OA

Inflammation is a key link in the occurrence and development of OA. Whether it is inflammation in the plasma or in the local soft tissue of the joint, it can cause OA. Studies have shown that stimulation of toll-like receptor (TLR) signaling can exacerbate invasive OA in mice (29). At the same time, serum high-sensitivity C-reactive protein (hs-CRP) levels were correlated with bone and joint WOMAC score and VAS score (31). Research has shown that, LPS and lipopolysaccharide-binding protein (LBP) were significantly associated with activated macrophages and osteophyte severity in the joints of Knee Osteoarthritis (KOA) patients (30). Guan et al. also reported that the main indicators of OA, bone volume over total volume (BV/TV), trabecular thickness (Tb.Th), and medial femoral condyle (MFC) were positively correlated with LPS, IL-6, and Tumor necrosis factor-α (TNF-α), and negatively correlated with the ratio of Firmicutes and Bacteroidetes (25). However, not all studies have shown a correlation between inflammatory markers and osteoarthritis. Studies have shown no statistically significant association between soluble Toll-like receptor 4 (sTLR4) or IL-6 and radiographic progression of OA (32).

## Discussions

Our systematic review suggests a link between low-grade inflammation caused by gut microbiota and osteoarthritis, but further research is needed in the future. Low-grade inflammation leads to OA through the production of inflammatory mediators, including innate immune activation, macrophage-dominated inflammatory response, Toll-like receptor (TLR) activation, and complement activation, among which TLR signaling plays an important role in the pathogenesis of OA (4, 36–38). Locally injured molecules activate TLRs, which trigger the secretion of pro-inflammatory substances and local inflammation in the joints (4, 38). It has been found that TLR expression is increased in areas of cartilage damage in OA patients (39). Upregulation of various TLR signaling components is seen in OA-associated chondrocytes, most notably LBP and cluster of differentiation 14 (CD14), which are accessory proteins of multiple TLRs and interact with multiple signaling molecules including LPS (37, 38).

Studies have shown that gut bacterial products such as LPS can enter the systemic circulation and affect many organs, including joints, by causing systemic low-grade inflammation (30, 40). LPS is an endotoxin associated with the outer membrane of various Gram-negative pathogens (41) and a classic innate immune system activator that activates host immune cells by binding to Toll-like proteins. Meanwhile, a correlation study between LPS and OA has shown that human serum LPS levels are associated with osteophyte severity in OA, and synovial fluid LPS is associated with osteophyte severity, joint space narrowing, and total pain/function severity scores (30).

Similar to LPS, LBP has also been shown to be associated with increased KOA severity in humans (30). LBP is mainly produced by hepatocytes and is a well-known acute phase reactant (42). LBP is activated by inflammatory mediators such as IL-6 and directly or indirectly by LPS itself (43–45). In humans, LBP triggers a dynamic endotoxin cascade by binding LPS and transferring it to CD14, which transfers LPS to the Toll-like receptor 4-myeloid differentiation protein-2 (TLR4-MD-2) receptor on immune cells; LBP thereby concentrates LPS on the cell membrane of immune cells, to induce an inflammatory response (46). LBP binds pro-inflammatory components of both Gram-positive and Gram-negative bacteria (47), making it a more prevalent marker of bacterial exposure than LPS derived only from Gram-negative bacteria (45). Meanwhile, other studies have shown that LBP is necessary for the inflammatory cascade triggered by saturated fatty acids and metabolic endotoxemia (48, 49).

A high-fat diet, an unhealthy dietary pattern that leads to obesity, altering microbial community structure and reduce microbial diversity, resulting in an increase in pro-inflammatory microbiota, thereby increasing intestinal permeability and circulating levels of LPS. In a high-fat diet model, TLR signaling plays a key role in low-grade inflammatory pathways (4, 50), such as toll-like receptor 4 (TLR4) (37, 51, 52), LPS, and LBP (31), and interleukin 6 (IL-6) (53–55), and have also been implicated in the inflammatory mechanisms of OA.

Exercise diversifies the gut microbiota and reduces the Firmicutes/Bacteroidetes ratio (56). This view was validated in our systematic review (28). At the same time, exercise produces high levels of endocannabinoids in arthritis patients, which mediate the gut microbiota to produce anti-inflammatory substances that reduce pain (57).

Gender variance is one of the factors affecting the prevalence of OA. A meta-analysis on global incidence and prevalence of OA in women is 1.69 and 1.39 times as much in males, respectively (58). Meanwhile, a study found that polymorphism in growth differentiation factor-5, estrogen-specific receptor-alpha, and calmodulin-1 has increased the disruption of cartilage and reduced mRNA and protein synthesis, which increased the risk of KOA in women (59). Moreover, A prevalence study on osteoporosis, hypovitaminosis D, and OA found higher rates of Vitamin D insufficiency and deficiency in women than in men (60), and there is a correlation between vitamin D deficiency and OA (61).

## Limitations

First, In the analysis of microbial sequencing, the analytical methods were different across studies involving various regions (V3-V5) and cut-off points for clustering OTUs which may affect the results. Second, the gut microbial community analysis by 16S rRNA sequencing was not used in all studies, which may affect the consistency of the results. Third, most of them are animal studies, and there are fewer extensive studies in humans, and fewer studies on the complexity of the gut microbiota and its association with OA. Finally, most studies have only observed changes in gut microbiota and inflammatory factors, but the underlying mechanisms have not been further explored.

## Conclusions

In conclusion, our systematic review provides evidence for the development of OA due to low-grade inflammation caused by intestinal flora disturbance. Further studies are needed to explore the mechanisms involved.

## Data availability statement

The original contributions presented in the study are included in the article/Supplementary material, further inquiries can be directed to the corresponding author/s.

## Author contributions

WX and HX conceived and designed research, performed experiments, and edited and revised manuscript. YJ analyzed data. BJ and YJ interpreted results of experiments. WX prepared figures and drafted manuscript. All authors contributed to the article and approved the submitted version.

## Funding

This study was supported by the Chongqing Beibei District Science and Technology Bureau Project (2022-18).

## Conflict of interest

The authors declare that the research was conducted in the absence of any commercial or financial relationships that could be construed as a potential conflict of interest.

## Publisher's note

All claims expressed in this article are solely those of the authors and do not necessarily represent those of their affiliated organizations, or those of the publisher, the editors and the reviewers. Any product that may be evaluated in this article, or claim that may be made by its manufacturer, is not guaranteed or endorsed by the publisher.
