# Supplementary material for: Association of low-grade inflammation caused by gut microbiota disturbances with osteoarthritis: A systematic review
Source: Front Vet Sci. 2022 Sep 12;9:938629. doi: 10.3389/fvets.2022.938629 (PMC9510893; doi:10.3389/fvets.2022.938629)
Supplement: Supplementary file 1 [file Table_1.DOCX]

**S1 Table.** CAMARADES CHECKLIST of the included studies

| **CAMARADES CHECKLIST** | **Ulici V 2018** | **Huang ZY 2020** | **Guan ZY 2020** | **Jhun JY 2021** | **Li KF 2021** | **Yoonkyung  2021** |
| --- | --- | --- | --- | --- | --- | --- |
| **Publication in peer-reviewed journal** | Y | Y | Y | Y | Y | Y |
| **Statement of control of temperature** | N | N | N | N | N | N |
| **Randomization of treatment or control** | N | N | N | N | Y | N |
| **Allocation concealment** | N | N | N | N | N | N |
| **Blinded assessment of outcome** | Y | Y | Y | Y | Y | Y |
| **Avoidance of anesthetics with marked intrinsic properties** | N | N | N | N | N | N |
| **Use of animals with hypertension or diabetes** | N | N | N | N | N | N |
| **Sample size calculation** | N | N | N | N | N | N |
| **Statement of compliance with regulatory requirements** | Y | Y | Y | Y | Y | Y |
| **Statement regarding possible conflict of interest** | Y | Y | Y | Y | Y | Y |
| **Total (on 10)** | **4** | **4** | **4** | **4** | **5** | **4** |
