# Supplementary material for: Association of low-grade inflammation caused by gut microbiota disturbances with osteoarthritis: A systematic review
Source: Front Vet Sci. 2022 Sep 12;9:938629. doi: 10.3389/fvets.2022.938629 (PMC9510893; doi:10.3389/fvets.2022.938629)
Supplement: Supplementary file 2 [file Table_2.DOCX]

**S2 Table**. SYRCLE bias assessment of the included studies

| **SYRCLE** | **Ulici V**  **2018** | **Huang ZY 2020** | **Guan ZY 2020** | **Jhun JY 2021** | **Li KF 2021** | **Yoonkyung  2021** |
| --- | --- | --- | --- | --- | --- | --- |
| **Was the allocation sequence adequately generated and applied?** | N | N | N | N | N | N |
| **Were the groups similar at baseline or were they adjusted for confounders in the analysis?** | Y | Y | Y | Y | Y | Y |
| **Was the allocation adequately concealed?** | N | N | N | N | N | N |
| **Were the animals randomly housed during the experiment?** | N | Y | N | N | N | N |
| **Were the caregivers and/or investigators blinded from knowledge which intervention each animal received during the experiment?** | N | N | N | N | N | N |
| **Were animals selected at random for outcome assessment?** | N | Y | N | N | N | N |
| **Was the outcome assessor blinded?** | Y | Y | Y | N | Y | N |
| **Were incomplete outcome data adequately addressed? (*)** | Y | N | N | N | N | N |
| **Are reports of the study free of selective outcome reporting? (*)** | Y | Y | Y | Y | Y | Y |
| **Was the study apparently free of other problems that could result in high risk of bias? (*)** | Y | Y | Y | Y | Y | Y |
| **Total (on 10)** | **5** | **6** | **4** | **3** | **4** | **3** |
