# Supplementary material for: Association of low-grade inflammation caused by gut microbiota disturbances with osteoarthritis: A systematic review
Source: Front Vet Sci. 2022 Sep 12;9:938629. doi: 10.3389/fvets.2022.938629 (PMC9510893; doi:10.3389/fvets.2022.938629)
Supplement: Supplementary file 3 [file Table_3.DOCX]

**S3 Table**. ROBINS bias assessment of the included studies

| **Author et al. (Year)** | **Study design** | **Risk of Bias** | **Inconsistency of results** | **Indirectness of evidence** | **Imprecision** | **Publication bias** | **Large magnitude of effect** | **Dose-response gradient** | **Plausible confounding** | **Quality** | **Confounding** | **Selection of participants into the study** | **Classification of interventions** | **Deviations from intended interventions** | **Missing data** | **Measurement of the outcome** |
| --- | --- | --- | --- | --- | --- | --- | --- | --- | --- | --- | --- | --- | --- | --- | --- | --- |
| Huang ZY (2016) | observational study | Low | Not serious | Not serious | Not serious | Not serious | N/A | N/A | No | Low | No information | Moderate | Low | No information | Low | Moderate |
| Boer CG (2019) | observational study | Low | Not serious | Not serious | Not serious | Not serious | N/A | N/A | No | High | Low | Low | Low | No information | Low | Low |
| Dunn CM (2020) | observational study | Low | Not serious | Not serious | Not serious | Not serious | N/A | N/A | No | High | Moderate | Moderate | Low | No information | Low | Low |
| Loeser RF (2021) | Case-control | Low | Not serious | Not serious | Not serious | Not serious | N/A | N/A | No | High | Low | Low | Low | No information | Low | Low |
|  |  |  |  |  |  |  |  |  |  |  |  |  |  |  |  |  |
