# Supplementary material for: Association of low-grade inflammation caused by gut microbiota disturbances with osteoarthritis: A systematic review
Source: Front Vet Sci. 2022 Sep 12;9:938629. doi: 10.3389/fvets.2022.938629 (PMC9510893; doi:10.3389/fvets.2022.938629)
Supplement: Supplementary file 4 [file Table_4.DOCX]

| **S4 Table**. Rob 2.0 bias assessment of the included studies | | | | | | | | | | | | | | | | |
| --- | --- | --- | --- | --- | --- | --- | --- | --- | --- | --- | --- | --- | --- | --- | --- | --- |
| **Author et al. (Year)** | **Study design** | **Risk of Bias** | **Inconsistency of results** | **Indirectness of evidence** | **Imprecision** | **Publication bias** | **Large magnitude of effect** | **Dose-response gradient** | **Plausible confounding** | **Quality** | **Randomization process** | **Deviations from intended interventions** | **Missing outcome data** | **Measurement of the outcome** | **Selection of the reported result** | **Overall** |
| Huang ZY (2018) | Secondary analysis of a clinical trial | Low | Not serious | Not serious | Not serious | Not serious | N/A | N/A | No | Moderate | Low | Low | Low | Low | Some concerns | Some concerns |
| Lei M  2017 | blinded-randomized clinical trial | Low | Not serious | Not serious | Not serious | Not serious | N/A | N/A | No | Moderate | Low | Low | Low | Low | Some concerns | Some concerns |
